# Prevalence and associations for symptoms of depression in patients with Parkinson’s disease: a Sri Lankan experience

**DOI:** 10.1186/s13033-016-0079-1

**Published:** 2016-06-16

**Authors:** Tharaka B. Herath, Milinda Withana, Chaturaka Rodrigo, Ranjani Gamage, Chanika Gamage

**Affiliations:** Institute of Neurology, National Hospital of Sri Lanka, Colombo, Sri Lanka; University Medical Unit, National Hospital of Sri Lanka, Colombo 10, Sri Lanka; Department of Clinical Medicine, Faculty of Medicine, University of Colombo, Colombo, Sri Lanka

**Keywords:** Parkinson’s disease, Depression, Sri Lanka

## Abstract

**Background:**

The prevalence and associations for depression in patients with Parkinson’s disease vary widely between studies. This reflects the influence of cultural, demographic and socioeconomic confounders within communities that make generalizations invalid. Therefore it is important to identify unique attributes within a community on this phenomenon. This is the first study from Sri Lanka on the prevalence and associations for co-morbid depression in patients with Parkinson’s disease.

**Methods:**

We conducted this cross sectional study at the Institute of Neurology, National Hospital of Sri Lanka. All patients with a diagnosis of idiopathic Parkinson’s disease followed up at the movement disorder clinic of the institute were enrolled. The patients were interviewed by investigators (medical practitioners) with an interviewer administered questionnaire that collected data on (a) demography, (b) clinical symptoms of Parkinson’s disease and (c) socioeconomic background. Symptoms of depression were assessed with Hamilton rating scale for depression.

**Results:**

We enrolled 75 patients [males; 54 (75 %), mean age; 63.6 years, SD ± 6.8]. Forty-six (61.3 %) patients had been either formally diagnosed with depression or showed symptoms of depression. Bradykinesia, monthly income below Rs. 10,000 and having a family history of depression were significantly associated with a diagnosis of life-time post Parkinson’s disease depression (p < 0.05).

**Conclusion:**

Given the potential benefit in treatment (for depression), all patients with Parkinson’s disease should be screened for depression regularly. HAM-D would be a good screening tool for this purpose as it has good reliability, validity and can be administered within a reasonable time limit.

## Background

Depression is a well-recognized complication of Parkinson’s disease (PD) [1]. The estimated prevalence of depression among patients with Parkinson’s disease vary widely among different studies (from 50 to 70 %) and it is difficult to point out a representative figure across different study cohorts [1]. Numbers are also influenced by other secondary factors such as family support, community support, severity of disease and individual life circumstances (e.g. financial stability, access to services) [2]. The severity of ongoing depression is also affected by receiving timely treatment for both PD and depression [3]. Therefore data on prevalence of depression and its associations cannot be generalized or extrapolated between communities. It is important to understand the social, cultural and health dynamics of individual populations to understand the impact of depression on patients with this debilitating neurological condition.

Parkinson’s disease is no longer an uncommon disease. With increased awareness of its existence among the lay community more people present to healthcare services and are diagnosed early. This is especially true in countries such as Sri Lanka where the demographic transition has resulted in an aging population. In the United States, approximately 1.6 % of the over 65 age group is affected by PD [4]. In Germany, the standardized prevalence of PD was 1680 cases per 100,000 of population and 32.6 % of them were suffering from depression [5]. The prevalence of disease reached a peak in patients older than 90 years. Data are not available for Sri Lanka, but in our experience, we are seeing more people with PD getting registered for follow up clinics. Overall, recent research on PD has shown that (a) in some patients, mood disorders and PD can have a common underlying neural mechanism [6], (b) mood and behavioural disorders can worsen the quality of life in patients with PD [7], (c) the prevalence of PD increases with age [5], (d) persistent depression can have an adverse effect in daily functioning of patients with PD [8] and (e) treatment can improve the functionality of patients with PD [3]. The management of depressed patients with PD should be a multidisciplinary effort not restricted to tackling the primary neurological problem of dopamine depletion. Understanding the prevalence of co-morbid depression and risk factors for depression in the local population with PD would enable clinicians to cater to the needs of patients better.

The objectives of this study were to (a) estimate the prevalence of depression (or depressive symptoms) among patients with PD presenting to a tertiary neurology clinic in the premier healthcare institution in Sri Lanka, (b) identify any significant clinical or demographic associations for presence of depression in this patient sample and (c) forward recommendations and suggestions for providing optimal care for patients with a dual diagnosis of PD and depression.

## Methods

We conducted this cross sectional study at the Institute of Neurology, National Hospital of Sri Lanka (NHSL). NHSL is the premier healthcare institution of the country and Institute of Neurology is its’ tertiary care referral centre for patients with neurological diseases. The institute has two units and unit 1 has a special clinic for movement disorder patients referred from hospitals from all over the country. All patients with a diagnosis of idiopathic PD followed up at the clinic were invited to take part in the study. Exclusion criteria were inability to give consent and impaired verbal expression. The need to exclude patients was decided on a case by case basis based on the judgement of clinicians.

All eligible consenting patients were interviewed by investigators (medical practitioners) with an interviewer administered questionnaire that collected data on (a) demography, (b) clinical severity of PD and (c) socioeconomic background. Symptoms of depression were assessed with Hamilton rating scale for depression (HAM-D) [9]. The HAM-D questionnaire lists 21 items but only 17 are scored. The first 8 items are scored on a scale of 0 (not present) to 4 (severe) while the other nine items are scored from 0 to 2. Of the final cumulative score, values of 8–13, 14–18, 19–22 and ≥23 indicates possibility of mild, moderate, severe and very severe depression respectively. Patients who had depression according to HAM-D were re-tested by a checklist for ICD-10 (International Classification of Diseases) criteria for depression.

The questionnaire was pretested on ten patients with PD followed up at Colombo South teaching hospital. The data were analysed with SPSS v15 statistical software, and significance of associations were calculated using Chi square test/Fishers exact test (for dichotomous data) and independent T test (for continuous data). Binary logistic regression analysis was carried out for all factors that were significant on bivariate analysis. Findings relevant to descriptive statistics were summarized into proportions and averages based on the scales of measurements.

### Ethical considerations

Ethical approval for the study was obtained from the ethics review committee of National Hospital of Sri Lanka. Informed written consent was obtained from patients prior to data collection. Personal information that could lead to identification of patient was not entered into the database.

## Results

Eighty-one patients were initially assessed for the study but six were excluded according to exclusion criteria. The final sample size was 75 patients [males; 54 (75 %), mean age; 63.6 years, SD ± 6.8]. Mean age of onset of PD in the sample was 55.8 years (SD ± 11.4 years). Other demographic and clinical characteristics of the sample are summarized in Tables 1 and 2 respectively.

**Table 1:** Demographic characteristics of participants (n = 75)

Characteristic	Number	Percentage (%)
Gender		
Male	54	72.0
Female	21	28.0
Civil status		
Married	72	96.0
Single	3	4.0
Employment		
Currently working	37	49.3
Retired/not working	38	50.7
Per capita income^a^ (LKR)		
Less than 10,000	22	29.3
10,001–30,000	46	61.3
30,001–45,000	7	9.3
More than 45,000	0	0.0
Level of education		
No formal education	3	4.0
Primary education	8	10.7
Secondary education	58	77.3
Tertiary education	6	8.0
Concurrent chronic medical problems^b^		
Diabetes	16	21.3
Hypertension	23	30.7
Ischaemic heart disease	9	12.0
Mental disorders	4	5.3
Other	1	1.3

^a^1 US Dollar = 133 Sri Lankan rupees (LKR) based on the exchange rate at the time of the study

^b^Self-reported co-morbidities

**Table 2:** Clinical characteristics related to Parkinson disease and depression among the participants

Characteristic	Number	Percentage (%)
Duration of Parkinson’s disease (years)		
Less than	14	18.7
1–5	33	44.0
6–10	20	26.7
More than	8	10.7
Symptoms		
Tremor	57	76.0
Bradykinesia	57	76.0
Postural instability	3	4.0
Rigidity	53	70.7
Formal diagnosis of depression after diagnosing Parkinson’s disease		
Yes	17	22.7
No	58	77.3
Presence of depressive symptoms according to HAM-D		
Yes	36	48.0
No	39	52.0
Severity grading of symptoms according to HAM-D score		
Mild	30	40.0
Moderate	5	6.7
Severe	0	0.0
Very severe	1	1.3
Family history of depression in first degree relatives	7	9.3
Family history of Parkinson’s disease in first degree relatives	7	9.3

The mean HAM-D score in the sample was 7.6 (SD ± 4.3, range 0–23). According to the scale, 36 (48 %) patients showed symptoms of depression (mild; 30, moderate; 5, very severe; 1). Of the patients that had no symptoms of depression according to HAM-D, 10 (25.6 %) had been diagnosed and treated for depression, after being diagnosed with PD. Of the patients who had symptoms of depression, only 7 (19.4 %) had been formally diagnosed and were on treatment at the time of evaluation. Others had not been diagnosed or referred to a psychiatrist. Overall 46 (61.3 %) patients in this sample had either been formally diagnosed with depression or showed symptoms of depression (previously undiagnosed) at the time of evaluation. For clarification, people who had been diagnosed with depression and were undergoing treatment at the time of study plus the patients who screened as positive for symptoms with HAM-D,will be referred to as patients with “ongoing depression or symptoms” (n = 36). “Life time post PD depression or symptoms group” includes all patients of the previous group plus the patients successfully treated of depression at the time of study (n = 46).

We assessed whether current age or age at diagnosis of PD showed a significant association for life-time post PD depression/symptoms. There was no such significant association (independent T test, p > 0.05). We also assessed whether any demographic, socioeconomic or clinical characteristics showed a significant association with ongoing depressive symptoms or life-time post PD depression/symptoms. The results are summarized in Table 3. Having bradykinetic symptoms, having an individual income below Rs. 10,000 and having a first degree relative with depression was significantly associated with life-time post PD depression or symptoms. Being currently employed was significantly associated with ongoing depression or symptoms (Chi square test, p < 0.05). When all significant variables on bivariate analysis were considered in a binary logistic regression analysis, both bradykinesia and lower individual income remained significant associations for life-time post PD depression or symptoms (Table 3). The predictive capacity of the regression model increased from 61.3 % (non model) to 74.7 % when these variables were included in the equation.

**Table 3:** Associations of demographic, socioeconomic and clinical variables and presence of depression or symptoms* in patients with Parkinson’s disease (PD), N = 75

Characteristic	Proportion with life time post PD depression or symptoms (%)^a^	Odd ratio (95 % confidence interval)	Adjusted odd ratio (95 % confidence interval)
*Sex*			
Male	34/54 (62.9 %)	1.27 (0.46–3.56)	–
Female	12/21 (57.1 %)	1	
*Level of Education*			
Less than grade 10	25/38 (65.7 %)	1.46 (0.56–3.73)	–
More than grade 10	21/37 (56.7 %)	1	
*Current employment* ^b^			
Yes	25/46 (54.3 %)	1.69 (0.66–4.32)	–
No	21/29 (72.4 %)	1	
*Monthly per capita income*			
Less than LKR 10,000	18/22 (81.8 %)	4.01 (1.12–13.48)^c^	4.05 (1.08–15.15)^c^
More than LKR 10,000	28/53 (52.8 %)	1	1
*Bradykinesia*			
Yes	16/18 (88.8 %)	7.20 (1.51–34.23)^c^	10.39 (2.08–51.72)^c^
No	30/57 (52.6 %)	1	1
*Tremor*			
Yes	34/54 (62.9 %)	1.28 (0.44–4.16)	–
No	12/18 (66.7 %)	1	
*Diabetes*			
Yes	10/16 (62.5 %)	1.06 (0.34–3.33)	–
No	36/59 (61.0 %)	1	
*Hypertension*			
Yes	11/23 (47.8 %)	0.44 (0.16–1.21)	–
No	35/52 (67.3 %)	1	
*Depression in a first degree relative*			
Yes	7/7 (100 %)	Infinity	Not applicable
No	0/68 (0 %)		
*Having PD for more than 5 years*			
Yes	32/47 (68.1 %)	0.47 (0.18–1.23)	–
No	14/28 (50.0 %)	1	

^a^Presence of symptoms of depression by HAM-D or having a formal diagnosis of depression was both considered together for analysis

^b^Employment (current) was significantly associated with ongoing depression or symptoms: OR 4.00 (1.53–10.46), p = 0.004

^c^p < 0.05

## Discussion

Depression in Parkinson’s disease is a well-recognized complication but data from developing countries are sparse. When treating the primary neurological condition aggressively, addressing the mental health needs of the patient is often missed. Unfortunately as shown by this study and other similar studies worldwide, depression is indeed a very common complication in patients with PD that must to be treated to improve the overall quality of life [1]. There are a multitude of circumstantial causes for low mood in a patient suffering from Parkinson’s disease such as loss of spontaneity, changes to physical appearance, lack of balance, speed and agility, dependence and concurrent physical illnesses related to disability (respiratory tract infections and hospitalizations). Whether these circumstantial factors alone can explain the high incidence of depression in PD is debated. More recent studies suggest that there may be structural neurological abnormalities (e.g. abnormal amygdala function) that puts a patient with PD at a higher risk than a healthy person for depression [6]. Interestingly a nationwide large scale study that assessed the long term health outcomes (over 5.5 years) in 174,776 individuals, concluded that being diagnosed with an anxiety disorder significantly increased the risk of a subsequent diagnosis of Parkinson’s disease (hazard ratio 1.38, 95 % confidence interval (CI) 1.26–1.51) [10]. There were 2258 incident cases of PD in this study making it one of the best studies on association of mental health and PD. Though this study excluded patients with depression from the analysis, the results show that there may be a biological link between mental health disorders and PD. With regard to depression, there is a similar population based follow up study from Sweden (n = 140,688) that showed a prior diagnosis of depression increases the risk of a subsequent diagnosis of Parkinson’s disease with a significant odds ratio of 3.2 (95 % confidence interval 2.5–4.1) in the first 12 months since the onset of depression [11]. The odds ratio dropped to 1.5 (95 % CI 1.1–2.0) after 15–25 years but still remained significant. There were 3260 incident cases of PD in this cohort. It was interesting to see whether patients in our sample were diagnosed with depression prior to the diagnosis of PD. However, all were diagnosed after the onset of PD. This may be because PD can increase the risk of developing depression and it is also easy for doctors to detect depression in individuals who are already in contact with the healthcare system due to their primary medical problem (PD).

Accurate diagnosis of depression in a patient with PD is a challenge given the mask like face, slowness and flat effect. There are studies that compared different tools to diagnose depression in this complicated clinical scenario. A head to head comparison of HAM-D and Montegomery-Asberg depression rating scale (MADRS) against a DSM-IV diagnosis of depression in patients with PD concluded that both tools were valid to diagnose depression in PD but that HAM-D was better [12]. Accordingly, we have used HAM-D for our study. The investigators also noted that with HAM-D, relatively higher scores (above 13) were more specific for diagnosis while lower scores were useful for screening.

There have been numerous associations to predict depression in PD. Disease severity, age of onset, gender and other demographic variables has been assessed but results have been inconsistent [13]. The immense heterogeneity observed in this regard may be explainable by the fact that mental illnesses are significantly influenced by a person’s socio-demographic and cultural milieu. Therefore observations in one country cannot be extrapolated to another. To serve the patients better it is important to have data and observations for local patients. Previous studies in other countries have noted that in some instances, sidedness of symptoms (right vs. left), gender and age of onset of PD to have a significant association with subsequent depression [14–16]. We could not duplicate these findings in our study. Errea et al. concluded that occurrence of depression was higher in younger patients with PD [15]. However, in a different study, Kostic et al. demonstrated that this significance disappears when corrected for the duration of illness [17]. In our analysis we also could not find any significant association for duration of PD and depression as well.

The factors that showed a significant association with presence of depression (or symptoms) in our study were; presence of bradykinesia, lower individual monthly income (less than LKR 10,000), being employed and having a first-degree relative with depression. In our opinion, this clearly shows the contribution of environmental circumstances for onset of depression in PD. Bradykinesia limits a patient’s mobility and capacity to fulfil activities of daily living. Such a patient is increasingly dependent on others for care. Similarly having an individual income below LKR 10,000 (75 USD) per month is considered inadequate (average per capita monthly income in Sri Lanka is LKR 12,000) [18]. However in most cases, the affected individual was a major contributor to the household finances and the illness would have caused a significant impact on total household income. Average household monthly expenditure in Sri Lanka as estimated in 2013 was LKR 41,000 [18]. Over the last 2 years it would have increased further. In addition, people who continued to work despite the illness were more likely to have depression (or symptoms) than those who were not employed. Pressing financial circumstances may necessitate patients to work despite their disability and increased awareness of disability when faced with daily stressors at workplace which could eventually lead to low mood and depression. Interestingly family history of depression was another risk factor that again could point to genetic as well as common environmental associations that drive the propensity for poor mental health.

Overall, depression or depressive symptoms were present in more than half (61.3 %) of our sample. More importantly, most of them were undiagnosed prior to screening in this study. Various treatment strategies have been tried for treating depression in PD but the mainstay of treatment are antidepressants and cognitive behaviour therapy. A meta-analysis of 893 PD patients enrolled over 20 randomized controlled trials have shown that both antidepressant treatment (specially the selective serotonin re-uptake inhibitors) and cognitive behaviour therapies to have a significant effect on improving depression [1]. In our sample also, 10 out of 17 individuals who had been diagnosed previously were completely free of symptoms when assessed with HAM-D. These findings underscore the importance of timely diagnosis and prompt referral for treatment of depression in patients with PD.

## Limitations

This study was limited to one treatment centre. However, it is noted that this is a tertiary care referral centre where patients get referred to from all over the country and probably the only centre in the country with a dedicated clinic for movement disorder patients. We did not compare the severity of depression with severity of PD as regular scoring of PD severity in form of United Kingdom Parkinson’s disease rating scale was not available. A one off measurement might have lead to inaccurate assessment of severity of disease as patients were on medication for PD. Depression was defined either as a formal diagnosis by a psychiatrist or by meeting the recommended cut-offs on HAM-D scale in which case it was referred to as depressive symptoms. Using HAM-D as a diagnostic tool has its limitations. Especially for scores that define mild depression, HAM-D is best described a screening tool rather than a diagnostic tool. This issue was partly offset by the fact that patient interviews and scoring of HAM-D was done by investigators who were also medical practitioners trained to diagnose depression. For patients who were positive on screening, we reconfirmed symptoms with a checklist of ICD-10 criteria for depression. Also, the fact that some PD features can mimic signs of depression could have interfered with accurate scoring of HAM-D.

## Conclusions

In this first study on prevalence of depression or depressive symptoms in a sample of PD patients followed up at a tertiary level neurological clinic in Sri Lanka, we found that a majority of patients (61.3 %) had either been diagnosed with depression or showed evidence of ongoing depression following a diagnosis of PD. In those treated for depression, most had a complete resolution of symptoms but the majority were undiagnosed. Bradykinesia, having an income below average per capita income and having a family history of depression in a first degree relative were significantly associated with life-time post PD depression or symptoms. Age of onset of PD, duration of PD, gender, level of education and prevalence of other co-morbidities such as diabetes and hypertension was not associated with this outcome. Given the potential benefit in treatment for depression, all patients with PD (and especially those with above risk factors) should be screened for depression regularly. HAM-D would be a good tool for this as it has good reliability, validity and can be administered within a reasonable time limit.

## Consent

Written informed consent was obtained from the patients for participation and publication of this study.
